# From the Hypotheses to Clinical Evidence in Retinal Therapy

**DOI:** 10.18502/jovr.v16i2.9092

**Published:** 2021-04-29

**Authors:** Bradley Beatson, J. Fernando Arevalo

**Affiliations:** Wilmer Eye Institute, Johns Hopkins University School of Medicine, Baltimore, MD

**Keywords:** Diabetic Macular Edema, Diabetic Retinopathy, Intravitreal Bevacizumab, Tractional Retinal Detachment, Vascular Endothelial Growth Factor, Vitrectomy

## Abstract

The off-label, therapeutic use of intravitreal bevacizumab (IVB) in vascular retinal diseases such as diabetic macular edema and proliferative diabetic retinopathy (PDR) has increased significantly due to its ability to reduce retinal neovascularization and slow progression of disease. Here, we will review the literature and investigative developments on the use of IVB as a preoperative adjuvant to vitrectomy in severe PDR, specifically focusing on its ability to reduce intra- and postoperative complications and its risk for progression or development of traction retinal detachment. In particular, this review will highlight the natural progression of evidence from case series and observations to prospective, randomized clinical trials.

##  INTRODUCTION

Proliferative diabetic retinopathy (PDR) is an advanced form of diabetic retinopathy and is a leading cause of new onset blindness in adults.^[1]^ It is a common complication of diabetes mellitus (DM) with an estimated global prevalence of 7% amongst the diabetic population.^[2,3]^ Diabetic retinopathy is characterized by increased retinal capillary permeability with leakage and occlusion, while PDR is the result of subsequent chronic retinal ischemia and refers to the development of neovascularization in the retina or optic disc. PDR can lead to complications such as neovascular glaucoma, vitreous hemorrhage, and tractional retinal detachment (TRD).

Elevated levels of vascular endothelial growth factor (VEGF) in the vitreous fluid have been associated with PDR and are a driving factor in the development of retinal neovascularization.^[4,5,6]^ The introduction of intravitreal anti-VEGF therapies such as bevacizumab (Avastin; Genentech Inc, San Francisco, CA), have thus offered a promising, less-invasive alternative to panretinal photocoagulation (PRP), the historical gold standard treatment for uncomplicated PDR. In clinical trials, the use of intravitreal anti-VEGF treatment has been shown to be non-inferior to PRP and effective in reducing retinal neovascularization in PDR.^[7,8,9]^ The use of anti-VEGF agents has also proven useful as adjuvant agents to vitrectomy, which is performed in cases of severe PDR or PDR associated with complications such as TRD.^[10,11]^ When used as adjuvants to vitrectomy, these agents are reported to improve clinical outcomes by reducing intraoperative bleeding, iatrogenic retinal breaks, and the incidence of recurrent vitreous hemorrhage.^[12]^ However, their utilization as adjuvants has also been associated with the progression or development of TRD in up to 5% of patients, which has resulted in a lack of consensus regarding the appropriateness of their use.^[13,14]^

This review discusses current literature on the use of anti-VEGF therapeutics, particularly intravitreal bevacizumab (IVB), as adjuvants to vitrectomy. In particular, this review will highlight the natural progression of evidence from case series and observations to prospective, randomized clinical trials.

### TRD Following IVB for PDR

In 2006, Chen and Park^[10]^ and Avery et al^[11]^ suggested that adjuvant IVB can help facilitate vitrectomy in severe PDR cases. A meta-analysis was later published and identified that IVB preoperative before vitrectomy resulted in significantly less intraoperative bleeding and frequency of endodiathermy (*p* < 0.01), less surgical time (*p* = 0.003), and improved final best-corrected visual acuity (BCVA) (*p* = 0.003) relative to vitrectomy alone. However, in 2008, we published a retrospective, multicenter case series of 211 eyes that received preoperative IVB before vitrectomy, with 11 of these eyes (5.2%) experiencing subsequent progression or development of TRD following IVB.^[13]^ Records were obtained from seven sites in the United States, Brazil, Argentina, Puerto Rico, Costa Rica, and Venezuela, and all patients had PDR refractory to PRP treatment that was performed at least two months prior to IVB. The mean time from IVB injection to TRD was 13 days (range, 3–31 days), which would be supportive of a cause–effect relationship. In this case series, most of the patients that developed TRD had poorly controlled DM with a mean HbA${}_{1c}$ of 10.6%, and all used insulin for glycemic control. Nine out of eleven (81.8%) developed TRD at least five days after the IBV injection, further suggesting that increased time from IVB to vitrectomy may lead to a higher incidence of TRD. However, a weakness of this retrospective case series was that the presence of TRD as a natural progression of severe PDR could not be ruled out, due to the absence of a control group.

In 2011, we published a follow-up to our initial data with a larger sample size, where we examined the incidence and risk factors associated with development of TRD in 698 eyes that had adjuvant IVB before vitrectomy for refractory PDR.^[15]^ In this study population, 25 (3.5%) eyes developed or had progression of a TRD following adjuvant IVB. The statistically significant risk factors for TRD identified in this study were a higher dose (2.5 mg vs 1.5 mg) of IVB (*p* = 0.022), more than 13 days from IVB to vitrectomy (*p* < 0.001), and a history of DM for more than 15 years at the time of IVB (*p* = 0.009). These findings were noteworthy, given that Avery et al^[11]^ had previously reported that diabetic eyes may be uniquely sensitive to IVB. These results also suggested that timely surgery should be prioritized following IVB administration, especially in eyes at risk of vision-threatening TRD progression into the central macular region. Furthermore, the use of low-dose IVB in eyes with pre-existing retinal traction would be recommended, given the increased frisk at higher doses. However, the findings of this study were similarly limited by its retrospective, nonrandomized, and uncontrolled nature.

### Prospective Analysis – Preoperative IVB Vs SHAM

These retrospective analyses were valuable in understanding the risk of developing TRD following preoperative IVB for vitrectomy and the risk factors associated with its occurrence, yet a controlled, prospective analysis was still needed to determine whether the effectiveness of IVB outweighed the risk of this complication. Consequentially, in 2019, we released the results of our prospective, randomized, and double-masked clinical trial investigating adjuvant IVB with vitrectomy for TRD in the setting of PDR in 224 eyes.^[16]^ This study was a multicenter study conducted by the Pan-American Collaborative Retina Study (PACORES) Group, taking place at 13 clinical sites in nine countries within Latin America, the United States, Saudi Arabia, and Spain. Enrolled patients were randomized in a 1:1 ratio to either vitrectomy plus IVB or vitrectomy plus sham (control arm). IVB administration occurred three to five days before surgery. Patients' baseline characteristics were similar with no statistical difference in age, gender, manner of glycemic control, or lens status.

The results showed that IVB had a significant positive effect on reducing intraoperative bleeding and reducing associated complications and iatrogenic retinal breaks. Specifically, it was found that 67.6% of eyes in the study group experienced intraoperative bleeding of any degree, while 89.2% of eyes in the control group experienced intraoperative bleeding (*p* < 0.001). Additionally, 31.3% of study eyes had grade-2 intraoperative bleeding versus 51.7% of control eyes (*p* = 0.004). The required use of endodiathermy occurred more frequently in the control group relative to the study group (66.9% vs 27.4%; *p* < 0.001), and the presence of at least one iatrogenic retinal break was also found more frequently in the control group (58.9% vs 34.3%; *p* = 0.001).

Relative to baseline, there was a statistically significant improvement in BCVA in both the study and control groups, although there was not a statistically significant difference between the two groups. At 12 months of follow-up, 73% of the study group achieved an improvement in two or more lines (10 letters) of ETDRS vision compared to 67.8% of the control group (*p* = 0.555). When the control and study groups were divided into subgroups according to the presence or absence of vitreous hemorrhage before surgery, there were significant increases in BCVA by final follow-up relative to baseline, but there were still no statistically significant differences between IVB and sham. Furthermore, the majority of eyes had retinas reattached with one procedure at the time of final follow-up at 12 months, with reattachment rates of 94.12% in the IVB group and 87.5% in the control group. This difference was not statistically significant (*p* = 0.097). TRD progression following injection was only seen in the IVB group, and occurred in three eyes (2.94%). However, despite the TRD progression, BCVA improved following vitrectomy in those three cases.

##  DISCUSSION

The use of IVB in the treatment of retinal diseases, including as an adjuvant to vitrectom, has long been controversial. Since first reports of its off-label use began in 2005, it has grown in popularity due to the much higher price of ranibizumab and aflibercept, the two VEGF inhibitors approved for ophthalmic disease.^[17,18,19]^ With the results of our prospective study, we believe that it is possible to save more eyes using preoperative IVB before vitrectomy in cases of severe PDR. The anti-neovascular effects of IVB have shown to reduce intra- and postoperative bleeding relative to a control arm and thus abate the incidence of iatrogenic retinal breaks and facilitate intraoperative fibroproliferative membrane dissection. While progression or development of TRD is a possibility in these cases, the risk can be managed using lower dose of IVB (1.5 mg), performing vitrectomy within four days of IVB administration, and considering judicious use in patients with a notably long history of DM.

##  SUMMARY

Both retrospective and prospective studies have shown the use of IVB to be effective in reducing intra- and postoperative complications when used as a preoperative adjuvant in vitrectomy for severe PDR, especially in repair of TRD. While there is a small risk (2.5–5%) of TRD progression or development following IVB administration, this risk can be managed by using low-dose IVB (1.5 mg) and performing surgery shortly following the administration of IVB.

##  Financial Support and Sponsorship

Unrestricted grant from Research to Prevent Blindness (Wilmer Eye Institute), Dr. Arevalo is the Edmund and Virginia Ball Professor.

##  Conflicts of Interest

There are no conflicts of interest.
